# Leptospira infection complicated by demyelinating disease: A case report

**DOI:** 10.3389/fneur.2022.1021364

**Published:** 2022-11-29

**Authors:** Shu-Xin Chen, Deng-Ke Han, Yin Liu, Zhi-Hua Ye, Kui Lu, Biao Xu, Hui-qiang Mai

**Affiliations:** ^1^Department of Emergency, Zhongshan People's Hospital, Zhongshan, China; ^2^Department of Laboratory Medicine, Zhongshan People's Hospital, Zhongshan, China; ^3^Department of Neurology, Zhongshan People's Hospital, Zhongshan, China

**Keywords:** leptospirosis, leptospira burgdorferi, multiple organ dysfunction, emergency, infection

## Abstract

Leptospirosis is a zoonotic disease, found worldwide, that is caused by bacteria of the genus *Leptospira*. People can be infected with *Leptospira* if they come in direct contact with the urine of an infected animal. Leptospirosis may be associated with demyelinating lesions of the central nervous system. This case report describes a 66-year-old female patient who presented with fever and generalized aches and progressed to unconsciousness within a few hours of admission. Laboratory tests showed *Leptospira* infection, and brain magnetic resonance imaging revealed acute demyelinating lesions. The patient responded well to penicillin and intravenous methylprednisolone therapy. Leptospirosis presenting with acute disseminated encephalomyelitis is rare. In this patient, an interdisciplinary collaboration involving the neurologist, radiologist, and pathologist was crucial for diagnosis and management. Further studies are warranted to investigate whether there is a correlation between demyelinating lesions and leptospiral infection.

## Introduction

In the past few decades, leptospirosis has become a significant infectious disease with high mortality (1). Yearly, an estimated one million people are infected with leptospirosis, and 5,000–6,000 people die (2). In both industrialized and developing countries, leptospirosis is a zoonotic disease affecting humans in rural and urban areas. Rats are the most common carriers of leptospirosis; those exposed to the urine of infected rats (e.g., through wounds) risk contracting it. Clinicians should therefore pay particular attention to any history of sewage exposure when taking a medical history. *Leptospira* bacteria directly damage tissues and immune-mediated mechanisms, leading to microcirculatory disorders as well as endothelial and organ dysfunction.

Leptospirosis often leads to severe complications such as acute kidney injury, liver dysfunction, myocardial involvement, and pulmonary hemorrhage (3). However, some atypical or unusual manifestations of leptospirosis, including ocular manifestations and neurological, hematologic, and gastrointestinal tract involvement, are often overlooked and rarely reported (4).

## Case report

The patient was a 66-year-old female farmer with a history of exposure to field sewage. In the absence of precipitating factors, she experienced general pain with fatigue and anorexia 3 days before coming to the emergency department. On the following day, she presented to a local doctor with shortness of breath and was treated with traditional Chinese medicine (no details available), but her symptoms did not significantly improve. She was then referred to the emergency department at the end of September 2021 with fever and dyspnea, and was admitted to the general ward.

Upon admission, her temperature was 37.1°C, pulse rate was 86/min, respiratory rate was 30/min, blood pressure was 96/50 mm Hg, and peripheral oxygen saturation (SpO_2_) was 90% in room air. Her physical examination was unremarkable. She had a previous medical history of hypertension and diabetes mellitus with poor blood glucose control. One hour after admission, her blood pressure dropped to 80/52 mm Hg, and her dyspnea increased. Eight hour after admission, her SpO_2_ fell to 78%. She lost consciousness and was transferred to the intensive care unit.

Laboratory investigations showed a white blood cell count of 11.09 × 10^9^/L with 87% neutrophils. Her interleukin-6 level was 208.6 pg/ml, and her procalcitonin was 3.8 ng/ml. Head CT on the 2nd day after admission revealed multiple lacunar cerebral infarctions in the bilateral corona radiata and right basal ganglia, mild brain atrophy, and intracranial arteriosclerosis; no other abnormalities were detected in the brain parenchyma. We performed a lumbar puncture on the 4th day after admission and sent the cerebrospinal fluid (CSF), along with her blood, for metagenomic next-generation sequencing (mNGS). Capillary blood glucose was 15.2 mmol/L at the time of lumbar puncture.

**Figure 1:**
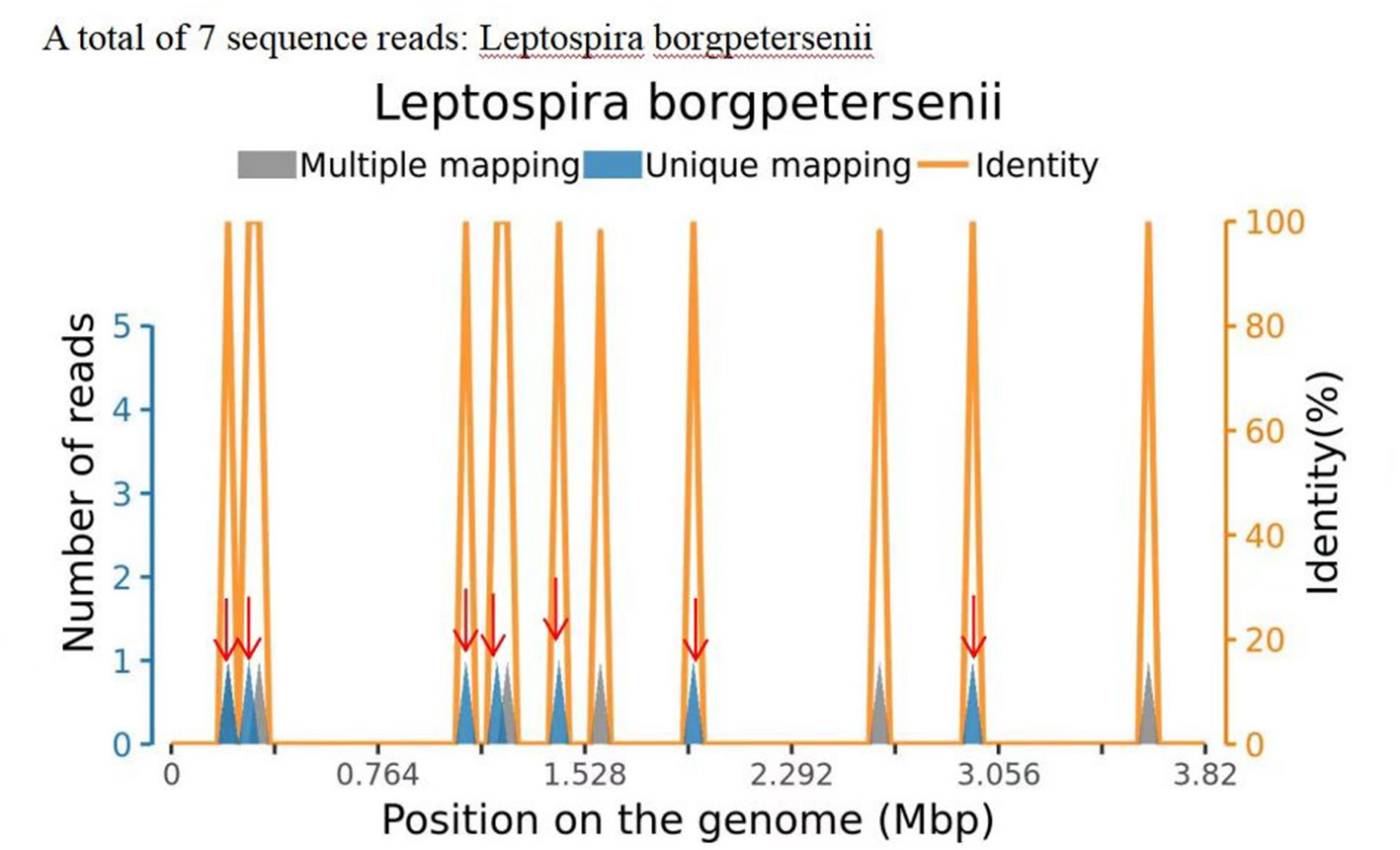
Sequence reads of *Leptospira borgpetersenii*, derived from the patient's cerebrospinal fluid (CSF) specimen and the *Leptospira* genome in the reference database. The coverage map shown above is a mapping for a specific microorganism, reflecting the distribution of the sequence of the microorganism on its genome; the abscissa represents the size of the microbe's gene group; and the ordinate represents the number of sequences detected in different genomic segments.

Information on the CSF workup is shown in Table 1. Organisms detected in the patient's CSF matched the *Leptospira* genomes in the reference database, identifying 7 sequencing reads of *Leptospira borgpetersenii* (Figure 1; Table 2). Table 2 also presents the details of the mNGS analysis. Likewise, high-throughput sequencing of her blood revealed the presence of *L. borgpetersenii*. In the meantime, the *Leptospira* IgG test was positive.

**Table 1:** Analysis of the cerebrospinal fluid workup.

**Item**	**Results**	**Reference range (units)**
Color	Light red	Colorless
Appearance	Slightly turbid	Clear
Clot	Nil	Nil
Cell count	2,000 ↑	0–8 (10 ^6/L)
WBC^*^ count	20 ↑	0–8 (10 ^6/L)
Glu	6.98 mmol/L	
Cl	141 mmol/L	120–132 mmol/L
Pandy's test	(±)	(–)
Upperlayer appearance	Colorless and clear	Colorless and clear
Underlayer appearance	Redness deposition^**^	
Multinucleate cell	Few leukocytes are not classified	%
Mononuclear cell	Few leukocytes are not classified	%

* WBC, white blood cell.

** Red blood cells (RBCs) are the result of puncture damage since MRI of the head did not indicate SAH. ↑ - Elevated.

**Table 2:** Results of the mNGS analysis.

**Type**	**Genus**	**Genus relative abundance (%)**	**Genus read number**	**Species**	**Identification confidence levels**	**Species read number**
pla	*Leptospira*	0.24	13	*Leptospira borgpetersenii*	0.99	7
G+	*Cutibacterium*	14.61	806	*Cutibacterium acnes*	0.99	626
G+	*Staphylococcus*	5.49	303	*Staphylococcus saprophyticus*	0.99	96
G+	*Staphylococcus*	5.49	303	*Staphylococcus epidermidis*	0.99	72
G+	*Staphylococcus*	5.49	303	*Staphylococcus hominis*	0.99	35
G+	*Corynebacterium*	3.70	204	*Corynebacterium accolens*	0.99	21
G+	*Micrococcus*	1.83	101	*Micrococcus luteus*	0.99	93
fun	*Candida parapsilos*	2.96	10	*Candida parapsilosis*	0.99	10

The blood collected for the mNGS was unfortunately misplaced by the third-party testing agency, so polymerase chain reaction validation could not be performed. No other pathogens were identified except *L. borgpetersenii*. The details of the confirmatory diagnostic testing for *L. borgpetersenii* are summarized in Table 3.

**Table 3:** Details of the confirmatory diagnostic testing for *L. borgpetersenii*.

**Collection date**	**Result date**	**Sample type**	**Testing method**	**Results**
2021-10-4	2021-10-7	Blood	mNGS	*L.borgpetersenii*
2021-10-4	2021-10-4	-	Head MR	acute demyelinating lesions
2021-10-5	2021-10-5	CSF	Biochemical analysis	Normal
2021-10-5	2021-10-8	CSF	mNGS	*L.borgpetersenii*
2021-10-8	2021-10-13	Serum	LEP-IgG antibody (ELISA method)	Positive
2021-10-13	2021-10-13	CSF	Biochemical analysis; AQP4 + MOG + MBP	Normal; negative

**Figure 2:**
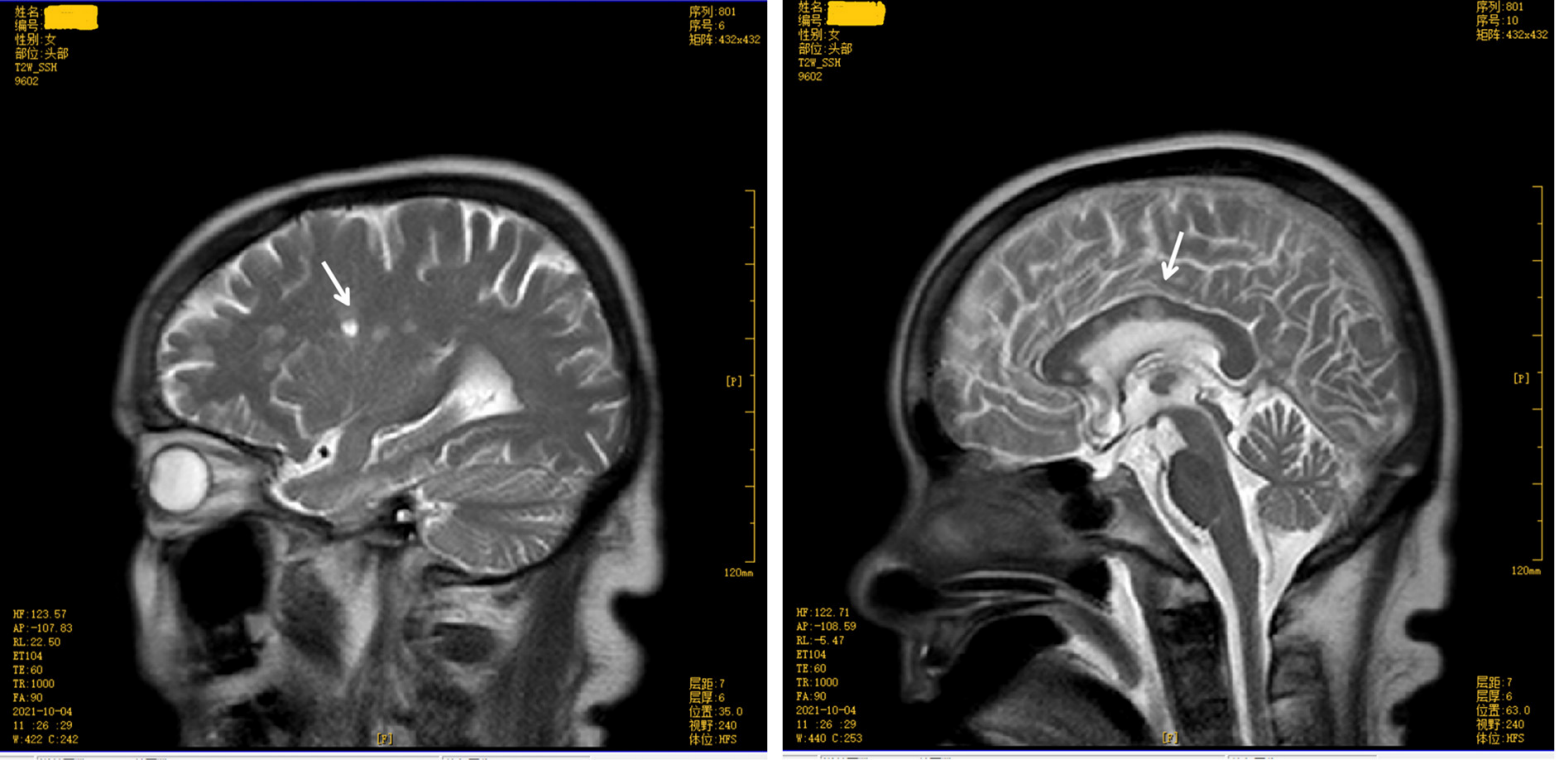
Brain magnetic resonance imaging (sagittal view) T2 weighted image shows multiple abnormal oval-shaped hyperintense signal lesions in the cerebral hemisphere and mesolobus.

**Figure 3:**
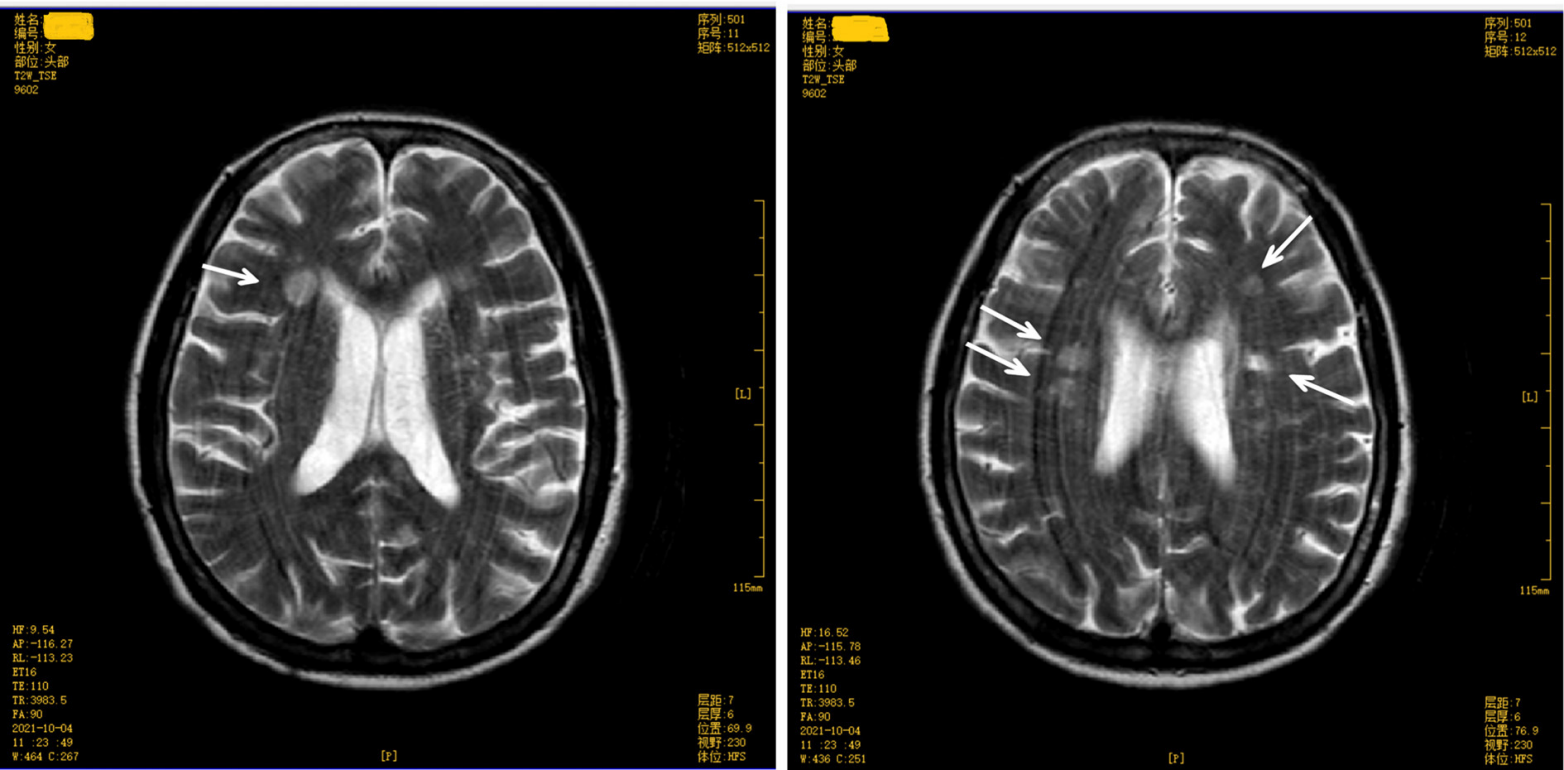
Brain magnetic resonance imaging (upper level of lateral ventricle) T2 weighted image shows multiple abnormal oval-shaped hyperintense signal lesions in the cerebral hemisphere and mesolobus.

The score on the Mini-Mental State Examination (education level: illiteracy) was 12. A magnetic resonance imaging (MRI) test of the brain revealed multiple symmetrical abnormal oval-shaped lesions with blurred boundaries in both cerebral hemispheres and the corpus callosum. A T2-weighted image (WI) showed hyperintense signals (see Figures 2, 3), and a T1 WI showed a hypointense signal. Accordingly, acute demyelinating lesions were considered. The patient's clinical presentation and results of the mNGS were consistent with a diagnosis of *Leptospira* infection and the MRI with acute- disseminated encephalomyelitis (ADEM).

Intravenous methylprednisolone pulse therapy (500 mg four times a day for 5 days) was started after the confirmatory diagnosis. The patient responded to treatment and was well oriented but still had some memory loss. The patient declined a repeat plain MRI for economic reasons. She was discharged after 3 months in the hospital, with her mental state restored to her preadmission level.

## Discussion

*Leptospira* are widespread and transmitted through skin abrasions (5). The nervous system is involved in about 10–15% of cases, and central nervous system (CNS) involvement is manifested chiefly as meningitis, encephalitis, and cerebral arteritis. The most common type of CNS involvement is aseptic meningitis. Most of the clinical features of neuroleptospirosis are due to capillary endothelial injury and vasculitis (6, 7). This patient's mental status rapidly worsened to coma within a few hours of admission. No lesions were found on plain spinal MRI, and an enhanced MRI scan was not performed.

In this case, a serological diagnosis by LEP-IgG followed by mNGS resources played a critical role in detecting *Leptospira* infection. The ELISA test is highly specific for detecting *Leptospira* antibodies, which peak in the blood after 2 to 3 weeks (8, 9). The mNGS, also known as high-throughput gene testing, is an attractive approach for pathogen detection that has facilitated the diagnosis, investigation, and tracking of infectious diseases (10). As early as 2014, Wilson et al. (11) reported a case of leptospirosis where the patient was finally diagnosed through high-throughput sequencing of CSF. High-throughput sequencing gave rapid results compared with other tests for leptospirosis (11). In this case, no other pathogens besides *L. borgpetersenii* were identified in the mNGS test or other tests. Given the patient's history of sewage exposure, a leptospirosis diagnosis was considered and subsequently confirmed.

Leptospirosis causes several types of nerve lesions, including mononeuritis, polyneuritis, polyradiculitis, myelitis, and cerebral arteritis. It is recommended that patients with leptospirosis presenting with neurological symptoms should undergo an MRI of the spine and brain, enhanced MRI scan, cerebral artery MRI, and digital subtraction angiography of the cerebral vessels (12). Brain magnetic resonance angiography (MRA) is helpful for the identification of leptospirosis-induced cerebral arteritis. However, a brain MRA was not performed on this patient.

Whether there is an overlap between demyelinating lesions and *Leptospira* infection needs further research. The spine MRI and neurological examination on admission showed no abnormality. The brain MRI showed acute demyelinating lesions. Demyelinating diseases are acquired and have different etiologies, but share some characteristics. The characteristic pathological changes are demyelinated nerve fibers seen in conjunction with relatively intact nerve cells.

ADEM is an immune-mediated demyelinating CNS disease. Its clinical features are multifocal neurological symptoms accompanied by neuroimaging evidence of multifocal demyelination (13). The disease mainly involves the brain and spinal cord, and is often secondary to infection or immunization. It was earlier believed that ADEM was caused by a viral infection (14).

The patient, in this case, had a sudden onset, confirmed infection, and neurological manifestations in the form of mental changes and cognitive impairment. A plain brain MRI revealed acute demyelinating lesions. The brain MRI scan showed multiple symmetrical abnormal oval-shaped lesions with blurred borders in both cerebral hemispheres and the corpus callosum, a high signal on T2 WI (see Figures 2, 3), and a low signal on T1 WI, suggesting acute-phase demyelination. The symptoms improved after high-dose active anti-infective steroid treatment. Thus, the diagnosis of ADEM (encephalitis type) was confirmed.

The incidence of neuroleptospirosis is ~0.86%. The pathogenesis of ADEM may be mediated by the activation of autoreactive lymphocytes (*via* a non-specific inflammatory process) that enter the CNS through a temporary breach in the blood–brain barrier (15). The activation of autologous T-cells leads to a transient autoimmune response to myelin sheaths and other autoantigens (16).

Leptospirosis mainly damages the microvascular endothelial cells, causing hemorrhagic vasculitis and microcirculatory dysfunction. The detailed pathogenesis of leptospirosis has not been fully explained to date. The adhesion and invasion of *Leptospira* into endothelial cells and metabolites, such as lipopolysaccharides and hemolysin, are believed to be responsible for the pathogenesis (17). In this patient, the possibility of infection through hidden wounds was considered, since there was a history of exposure to sewage in the field.

The intersections of CNS damage caused by ADEM and *Leptospira* infection require further study. The correlations between acute demyelinating disease and differences in *Leptospira* type, virulence, quantity, and individual reaction remain unknown. Patients with *Leptospira* infection complicated by CNS damage should alert the physician to the possibility of secondary demyelinating lesions. A brain MRI is essential for its diagnosis (18). Once an acute demyelinating syndrome is diagnosed, treatment should be aimed at reducing inflammation and immune activation as soon as possible to reduce the duration and severity of the disease. Treatment methods include high-dose intravenous corticosteroids, therapeutic plasma exchange, and intravenous immunoglobulins (19). This patient was treated with high-dose intravenous methylprednisolone.

For a precise diagnosis of demyelinating diseases, other relevant examinations are necessary (i.e., ADEM and multiple sclerosis are both inflammatory demyelinating diseases that should be differentiated). The electrophoretic test for oligoclonal bands in the CSF has diagnostic value for inflammatory diseases of the CNS, in particular, Guillain–Barre syndrome and multiple sclerosis (20). Unfortunately, this test was not performed on this patient.

This case report describes a patient with ADEM due to leptospirosis. This case report focuses on the diagnosis and treatment in a non-specialty hospital of an atypical presentation of secondary CNS damage attributable to leptospirosis. ADEM secondary to *Leptospira* infection requires differentiation from leptospiral cerebral arteritis. This case report focuses on the atypical presentation of secondary nervous system damage following leptospirosis. In this patient, mNGS provided a reliable method for diagnosing this complex case of leptospirosis and ensuring timely and effective treatment.

## Limitation

Over all, this case has several limitations. First, the quality of clinical management can be improved. When the patient was admitted for fever, the risk associated with the patient being a farmer was ignored, and only empirical antibiotics were given. An mNGS is not easy to obtain. Second, the necessary medical tests for the differential diagnosis, such as OB and MRA, were not done. In addition, due to economic considerations, patients may not complete necessary examinations from physician recommendations during treatment and follow-up, which may present challenges for clinicians.

## Conclusion

In patients with *Leptospira* infection exhibiting CNS symptoms, acute demyelinating disease and cerebral arteritis should be considered in the differential diagnosis. For patients with demyelinating disease, as indicated by imaging results, CSF should be examined for anti-aquaporin-4 antibody, anti-myelin oligodendrocyte glycoprotein antibody, anti-myelin basic protein antibody, and oligoclonal bands to further clarify the diagnosis. At present, the pathogenesis of *Leptospira* infection remains unclear. Conducting multidisciplinary consultations for such patients with the assistance of the infectious disease, neurology, radiology, and pathology departments will significantly benefit patients in this regard.

## Data availability statement

The original contributions presented in the study are included in the article/supplementary material, further inquiries can be directed to the corresponding author.

## Ethics statement

The studies involving human participants were reviewed and approved by Zhongshan People's Hospital Ethics Committee (Approval number 2022-026). The patients/participants provided their written informed consent to participate in this study.

## Author contributions

S-XC and D-KH conceived the idea and conceptualized the study. YL and Z-HY collected the data and analyzed the data. KL and BX drafted the manuscript. H-qM reviewed the manuscript. All authors have read and approved the final draft.

## Conflict of interest

The authors declare that the research was conducted in the absence of any commercial or financial relationships that could be construed as a potential conflict of interest.

## Publisher's note

All claims expressed in this article are solely those of the authors and do not necessarily represent those of their affiliated organizations, or those of the publisher, the editors and the reviewers. Any product that may be evaluated in this article, or claim that may be made by its manufacturer, is not guaranteed or endorsed by the publisher.
